# Mining Gene Expression Data of Multiple Sclerosis

**DOI:** 10.1371/journal.pone.0100052

**Published:** 2014-06-16

**Authors:** Pi Guo, Qin Zhang, Zhenli Zhu, Zhengliang Huang, Ke Li

**Affiliations:** 1 Department of Public Health, Shantou University Medical College, Shantou City, Guangdong Province, China; 2 Good Clinical Practice Office, Cancer Hospital of Shantou University Medical College, Shantou City, Guangdong Province, China; 3 Laboratory of Cell Senescence, Shantou University Medical College, Shantou City, Guangdong Province, China; University of Jaén, Spain

## Abstract

**Objectives:**

Microarray produces a large amount of gene expression data, containing various biological implications. The challenge is to detect a panel of discriminative genes associated with disease. This study proposed a robust classification model for gene selection using gene expression data, and performed an analysis to identify disease-related genes using multiple sclerosis as an example.

**Materials and methods:**

Gene expression profiles based on the transcriptome of peripheral blood mononuclear cells from a total of 44 samples from 26 multiple sclerosis patients and 18 individuals with other neurological diseases (control) were analyzed. Feature selection algorithms including Support Vector Machine based on Recursive Feature Elimination, Receiver Operating Characteristic Curve, and Boruta algorithms were jointly performed to select candidate genes associating with multiple sclerosis. Multiple classification models categorized samples into two different groups based on the identified genes. Models’ performance was evaluated using cross-validation methods, and an optimal classifier for gene selection was determined.

**Results:**

An overlapping feature set was identified consisting of 8 genes that were differentially expressed between the two phenotype groups. The genes were significantly associated with the pathways of apoptosis and cytokine-cytokine receptor interaction. TNFSF10 was significantly associated with multiple sclerosis. A Support Vector Machine model was established based on the featured genes and gave a practical accuracy of ∼86%. This binary classification model also outperformed the other models in terms of Sensitivity, Specificity and F1 score.

**Conclusions:**

The combined analytical framework integrating feature ranking algorithms and Support Vector Machine model could be used for selecting genes for other diseases.

## Introduction

As powerful tools for facilitating the discovery of totally novel and unexpected functional roles of genes, gene expression microarrays have been applied to a range of applications in biomedical research and produce a large number of databanks containing various amounts of hidden biological information [1]. The key resides in the ability to analyze large amounts of data to detect a panel of genes capable of discriminating diseases. This study proposed a modeling framework for establishing a robust classification model, for identification of disease-related genes. We utilized the proposed modeling approach for identification of genes involved in multiple sclerosis.

Multiple sclerosis is characterized as an inflammatory disorder of the central nervous system in which focal lymphocytic infiltration leads to damage of myelin and axons [2]. The trigger for multiple sclerosis is unclear so far, although it is generally evaluated as an autoimmune disease [3]. At present the diagnosis of multiple sclerosis usually involves the tests of lumbar puncture or magnetic resonance imaging scan of the brain function. The diagnostic ways are either clinically invasive or expensive for multiple sclerosis patients. High throughput technique of microarray has been applied to measure gene expression patterns of multiple sclerosis, and the challenge is to develop more effective approaches to identify a panel of genes that go beyond over-or-under expressing genes from the big data. In this study we reanalyzed the microarray dataset of multiple sclerosis from Brynedal et al. [4] using data mining methods, and selected discriminative genes. The computationally intensive methods of data mining provide us an effective way to rank features, allowing a careful selection of feature sets for optimal classification fitting. Therefore, we were able to investigate some genes with potential biological implications from microarray data. The aim of this study was to build a robust classification model with characteristics of feature selection and sample prediction.

Prior studies showed that combinatorial gene selection methods could be effectively applied to identify the gene signature for disease [5]. Zhou et al. [6] conducted a union method combining two feature selection algorithms, and identified significant risk factors for osteoporosis from a very large amount of candidates. This work introduced a combinational strategy to predict multiple sclerosis samples using microarray data. In the initial stage, a feature selection algorithm was used to extract the biologically-interpretable genes. A combined approach integrating three feature selection algorithms including Support Vector Machine based on Recursive Feature Elimination (SVM-RFE) [7], Receiver Operating Characteristic (ROC) Curve [8], and Boruta [9] was performed to rank genes, and order genes based on their importance. Then, an overlapping set of genes was selected. The SVM-RFE algorithm can eliminate gene redundancy automatically, retain a better and more compact gene subset, and yield a better classification performance. The ROC algorithm is to characterize a best separation between the distributions for two groups, and is easy to implement. The Boruta algorithm measures the importance of each feature. These three feature selection algorithms had high performance in learning, and their outputs were easy to understand.

We constructed six classical models including SVM, Random Forests, naïve Bayes, Artificial Neural Network, Logistic Regression and k-Nearest Neighbor to predict samples based on the feature subset. These models are widely employed in gene classification and have practical predicting performance. We introduced these techniques to classify the samples, evaluated them using cross-validation methods, and then utilized the optimal model to construct a gene selection model. As evaluated by several statistical metrics, an optimal SVM model was proposed, and it has shown to be useful for selecting disease-related genes in multiple sclerosis.

## Materials and Methods

**Figure 1:**
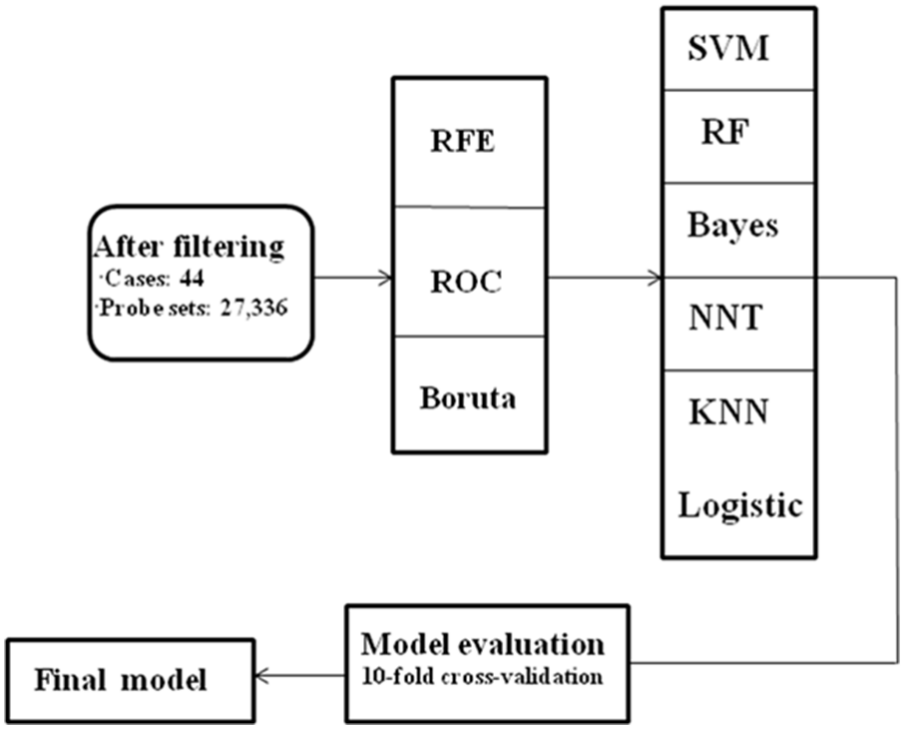
Flow chart of data analysis. The four major steps of this study: data preprocessing, feature selection, model building, and performance validation.

The process of data collection and analysis is illustrated in Figure 1, and the details of each step can be found in the following subsections.

### Data Collection and Processing

Gene expression profiles for a total 44 subjects were obtained from the ArrayExpress Database under accession number of E-MTAB-69. Accordingly, global gene expression in peripheral blood mononuclear cells samples was assessed in 26 multiple sclerosis patients. For the control, a population consisting of 18 individuals with other neurological diseases was also examined to assess their specific expression profiles of multiple sclerosis. The transcripts of peripheral blood lymphocyte were hybridized individually to the Human Genome 133 plus 2.0 arrays (Affymetrix, Santa Clara, CA) platform according to standard operating protocols. A full description of experimental protocols and processes can be viewed in the study conducted by Brynedal et al. [4].

The raw fluorescence intensity data were converted to gene expression values using the Robust Multichip Analysis algorithm [10] in Expression ConsoleTM Software. Each expression profile containing 54, 675 probe sets was preprocessed including background correction, normalization and summation of the intensities for each sample [10]. Probes with less discriminative power were removed according to the measurement of overall variance, which was implemented with the varFilter function using the genefilter package from the Bioconductor project [11] within R software [12]. After the preprocessing, a total of 27, 336 probe sets from each sample were used for further analysis.

### Feature Selection

#### SVM-RFE algorithm

The idea of the SVM-RFE algorithm is to use the weight magnitude of the SVM classifier as a feature ranking criterion to produce a feature ranked list [7]. The SVM-RFE algorithm is defined as the iterative three steps:

- Train the SVM;
- Compute the ranking criterion 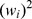 for all features based on the weight vector 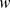;
- Remove the feature with smallest ranking criterion.

When all the iterative procedures have finished, a feature ranked list 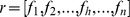 is obtained according to the evaluations for features.

#### ROC algorithm

The ROC curve is a particularly suitable and effective method to rank genes in regards to differential expression between tissues [8]. Suppose that 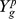 and 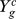 respectively represent the distributions of two phenotype groups for gene 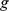. The idea of the ROC algorithm is to characterize separations and find a best one between the distributions for 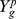 and 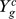.

Then, the partial area under the curve 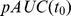 and the area under the entire curve 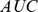 are commonly used to rank genes for differential expression in tissue samples. These two statistical measures are defined as equation (1) and (2):
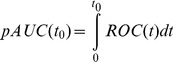
(1)

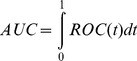
(2)where 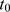 is some small false positive rate. Differentially expressed genes can be ranked based on the results of 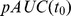 and 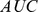.

#### Boruta algorithm

The Boruta algorithm is designed to iteratively remove the features which are proven to be less relevant than random probes [9]. The random forest classification algorithm runs fast without usually tuning parameters and it gives an estimate of feature importance [13]. Briefly, it is an ensemble of tree predictors in which each tree depends on the independent identically distributed random vectors in the forest.

In the Boruta algorithm, shadow attributes for the original attributes are created by shuffling values of the original attributes across objects, and thus the importance of shadow attributes is estimated and used as a reference for deciding truly important attributes. By adding randomness into the model system and correcting for the random fluctuations based on the ensemble of extra randomness, the Boruta algorithm aims to determine which attributes are truly important. The Boruta algorithm was implemented using the Boruta package [9] within R.

### Data Encoding and Feature Selecting

To encode microarray data to be fed into the feature selection step, the gene expression values were used to construct a gene expression matrix *M*, which was composed of 27,336 rows representing probe sets in each gene expression profile and 44 columns representing samples. A *Y* vector was generated to represent grouped statuses of each sample with ″0″ denoting the “control group” and ″1″ denoting the “multiple sclerosis group”. Then, the matrix *M* and the vector *Y* were input into the feature selection algorithms, which iteratively evaluated a candidate subset of features using the grouping information of samples, and generated a satisfactory feature subset. Due to two different kinds of feature selection algorithms (i.e., SVM-RFE and ROC algorithms rank genes in order, but Boruta algorithm directly generates a subset of genes with the label of “important”) used in this study, we selected the top 1,000 results from each SVM-RFE and ROC algorithms, and the output genes with the label of “important” were chosen for the Boruta algorithm. In fact, the Boruta algorithm only generated a subset of significant genes from a pool of candidates. To determine the significant category of genes in the SVM-RFE and ROC algorithms as done by Boruta algorithm, the moderated *t*-test was applied to test the statistical significance of the top percentage of genes in the two algorithms.

### Gene Function Analysis

Initially, each probe set from feature selection algorithms was mapped to an annotation of Entrez Gene ID and full gene name using the GeneCards Human Gene Database (http://www.genecards.org/). It is an integrated system that provides concise genomic related information, on all known and predicted human genes. GeneCards also gives out the counts of already reported studies as strength indicator of association between genes and potential diseases. We submitted gene symbols into GeneCards, and attempted to evaluate the associations. In the next phase, the Kyoto Encyclopedia of Genes and Genomes (KEGG) pathway enrichment of the identified genes was assessed using the database of Gene Annotation Tool to Help Explain Relationships (GATHER) (http://gather.genome.duke.edu/). GATHER is a proposed bioinformatics tool that can integrate various available data to extract the full value from molecular signatures produced from high-throughput assays [14]. We also performed a Gene Ontology enrichment analysis of genes based on GATHER. The GATHER system annotates genes with functional descriptors from Gene Ontology, and quantifies the significance of functional associations with a group of genes. The significance of association between a gene group and an annotation was assessed using a Bayes factor. The larger magnitude represents the stronger the functional association [14]. The *P*-values were statistically corrected for multiple testing using the Bonferroni method in this study. The limma package in R software was used to perform the moderated *t*-test to investigate the differential expression of the selected probes between multiple sclerosis patients and controls.

### Classification Models Building and Assessing

Three feature selection algorithms were conducted to rank genes according to the algorithms’ scorings. Each gene was ranked based on its prediction performance in each algorithm. After that three ranked gene sets were generated respectively, and an overlapped gene set was finally determined. Multiple classification models including SVM, Random Forests, naïve Bayes, Artificial Neural Network, Logistic Regression and k-Nearest Neighbor were established using the MLInterfaces package in the R software. The 10-fold cross validation method was performed to assess the prediction accuracy of each classifier. The 10-fold cross-validation is an effective method to evaluate the performance classification models [15]. The principle of this approach is to randomly partition the original sample into ten subsamples. Of these subsamples, one single subsample is retained as the validation dataset for testing the model, and the remaining subsamples are used for training data. The process is repeated 10 times, and the results are averaged to produce a final estimation of performance.

In a classification model, each sample was predicted into one of the two groups, i.e. multiple sclerosis subjects and controls. We applied the statistical measures of Sensitivity, Specificity, Accuracy and F1 score [16] for performance evaluation. The measures were defined as follows:
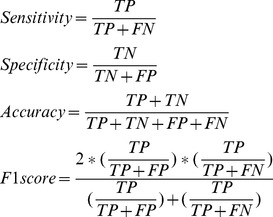
(3)where *TP* is the number of true positives, *FN* is the number of false negatives, *TN* is the number of true negatives and *FP* is the number of false positives.

## Results

### Ranking Genes of Multiple Sclerosis

**Figure 2:**
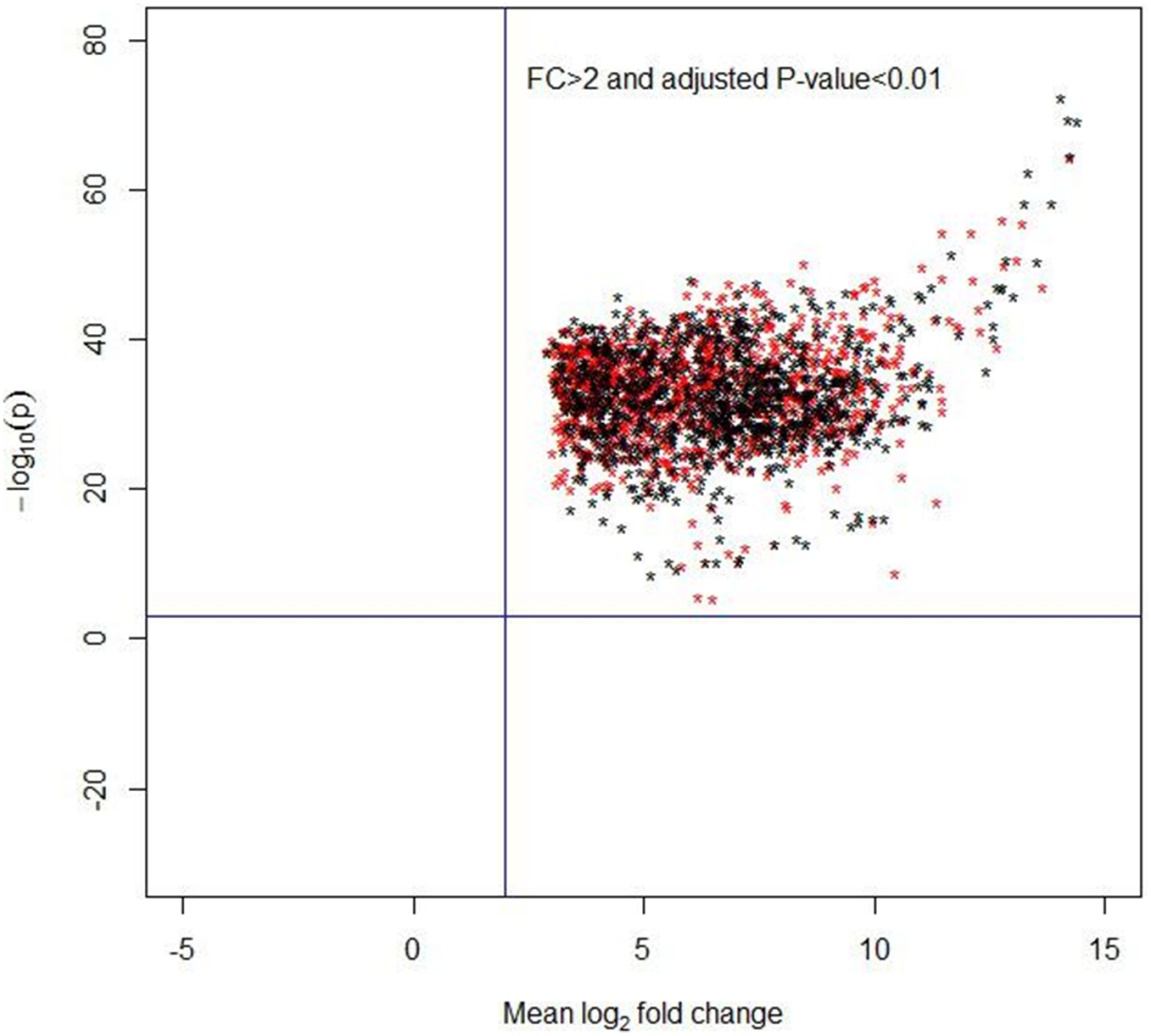
Analysis results of gene expression of the top 1000 genes selected from SVM-RFE (red symbol of star) and ROC (black symbol of star) algorithms. Genes with log fold change (FC) of expression >2 and adjuste *P*-value <0.01 were in the upper right area.

**Figure 3:**
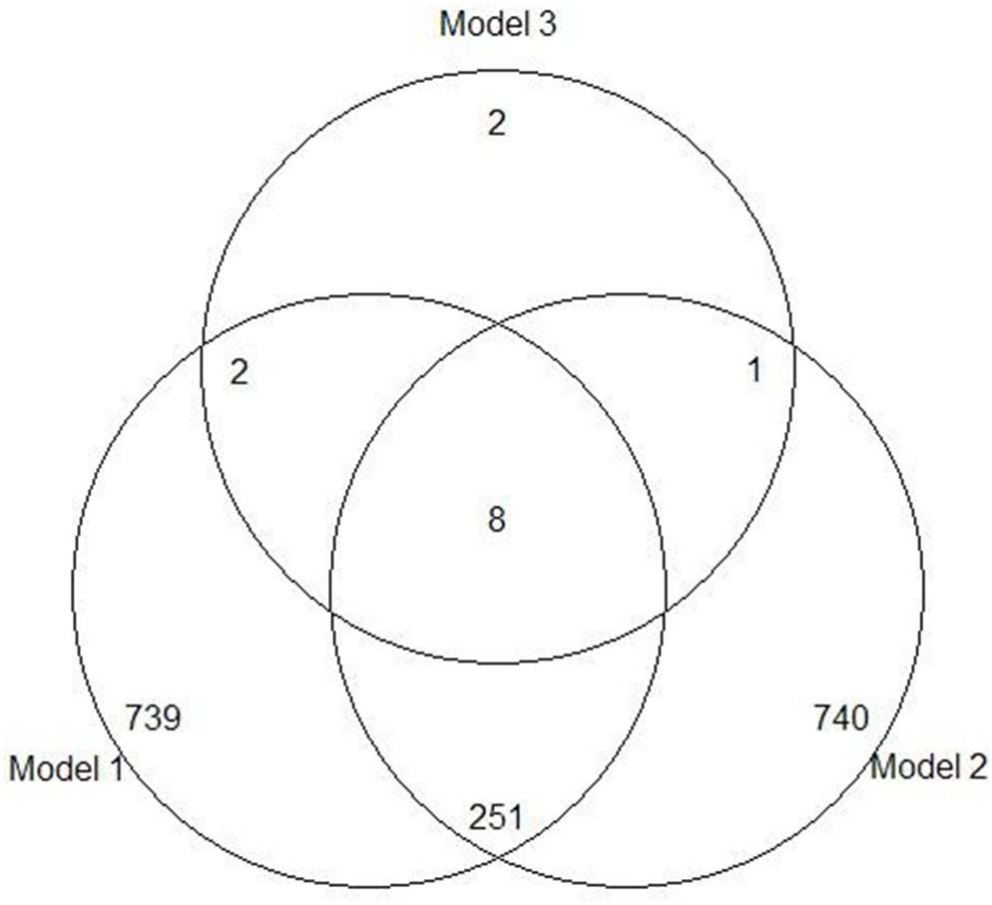
Overlapping features based on the ranked feature sets generated by three algorithms. Model 1: Support Vector Machine based on Recursive Feature Elimination (SVM-RFE) algorithm; Model 2: Receiver Operating Characteristic (ROC) Curve algorithm; Model 3: Boruta algorithm. In this procedure, an overlapping set, including 8 features, was identified and used for gene matching.

The SVM-RFE, ROC and Boruta algorithms were performed to rank all 27, 336 probe sets for each participating subject. Due to the Boruta algorithm outputting a subset of probe sets with the “important” lable, we chose the “important” probe sets and ordered the probe sets by the Z-score which indicating the measure of feature importance. However, the SVM-RFE and ROC algorithms directly ranked the probe sets in a sequence set. To determine the significant category of genes in the SVM-RFE and ROC algorithms as done by Boruta algorithm, the moderated *t*-test was applied to obtain the significant genes (Figure 2). Based on the analysis results of the adjusted *t*-test, both two sets of the top 1,000 genes were all significant, and their expressions of log fold change between the twop groups were more than 2 (Figure 2). Hence, three important sets of genes were integrated and their overlapping genes were investigated. A Venn diagram was used to similarly represent the intersection of the three sequence sets. The top significant genes from SVM-RFE and ROC algorithms and the important ones from Boruta algorithm were used to determine the overlapping genes (Figure 3). There were a total of 8 genes indicating the top hits from the three algorithms in the intersection of the diagram, and the expression values of these genes for each subject were used as the input variables in the classification models.

### Discriminative Ability of each Gene

**Figure 4:**
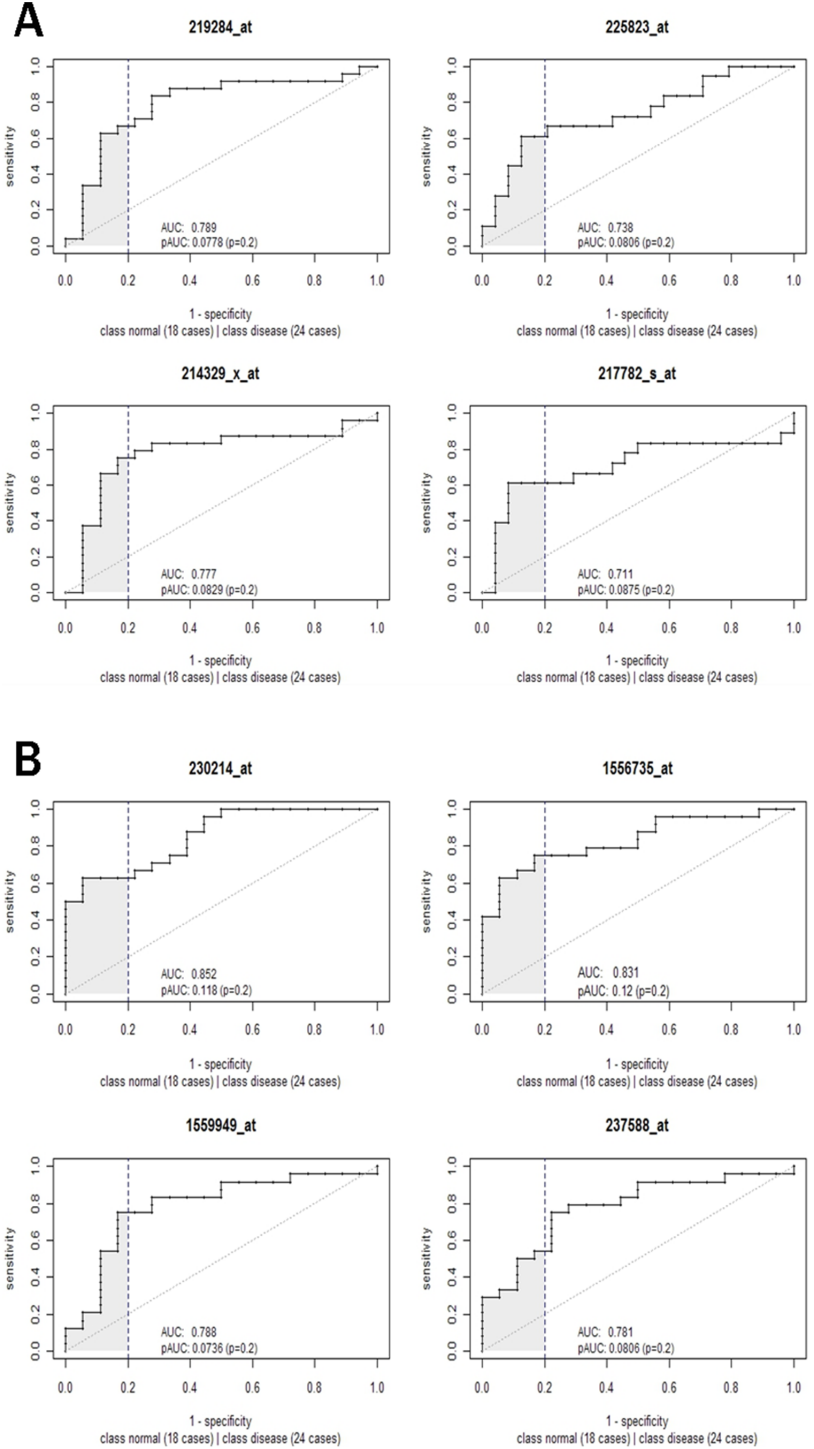
Receiver operating characteristic (ROC) curves for evaluating identified features. AUC (area under the curve) and pAUC (partial area under the curve) indicators were computed to assess the performance for each feature.

We visualized the expression profiles of the 8 genes in all 44 samples using ROC curves to illustrate the discriminative power between the two classes of samples for each gene (Figure 4). The indicators including pAUC (partial area under the curve) and AUC (area under the curve), were computed to assess the performance for each feature. The variation of AUC of the 8 genes ranged from 0.711 (probe 217782_s_at) to 0.852 (probe 230214_at), and 6 of them had AUC>0.78. Both the AUC and pAUC measures suggested the features held good classification performance.

**Figure 5:**
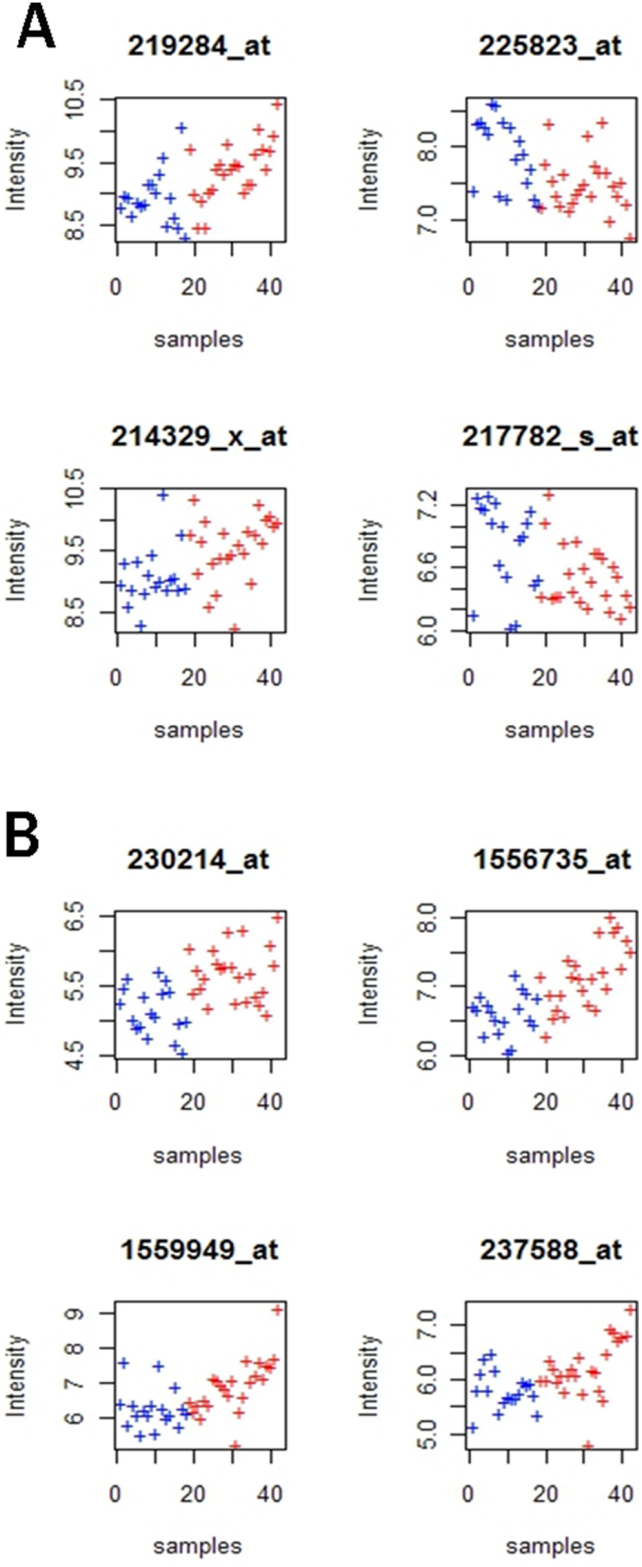
Scatter plot of expression values of eight features. Each panel in the above plot corresponds to one probe set. The y-axis represents the logarithmic expression intensity of each probe set, and the x-axis represents the samples. The red and blue colors respectively represent the multiple sclerosis and control groups.

A scatter plot for the 8 genes was also used to illustrate their discriminative power between the two classes of samples (Figure 5). Each panel in the plot corresponds to one feature gene, and different expression levels of these genes between the two groups can be observed. According to the scatterplot, these 8 genes clearly showed differential expression between multiple sclerosis patients and controls, supporting the ability of these genes to differentiate between individuals with and without multiple sclerosis.

### Gene Ontology and KEGG Pathway Enrichment Analysis

Each selected probe set was mapped to an annotation of Entrez Gene ID and the full gene name using the GeneCards database (Table 1). These 8 selected probes showed significantly differential expression between multiple sclerosis patients and controls (all adjusted *P*-values<0.05), and their log fold change were consistently greater than 2 (Table 1). The KEGG enrichment analysis (Table 2) revealed that the identified genes were closely related to apoptosis and cytokine-cytokine receptor interaction pathways (all adjusted *P*-values<0.05). TNFSF10 were suggested to be potentially associated with multiple sclerosis. In the Gene Ontology enrichment analysis, differentially expressed genes in multiple sclerosis subjects versus controls mainly involved protein kinase cascadse, inactivation of MAPK, regulation of signal transduction and apoptosis (Table S1 in File S1.). Differentially regulated genes primarily included TNFSF10, GPS1 and TRPS1. The information retrieved from GeneCards showed there were six published studies reporting on the relationship between TNFSF10 and multiple sclerosis (Table S2 in File S1.).

**Table 1:** Annotations of gene symbol and full gene name for each selected probe set and the differential expression analysis using moderated *t*-test.

Porbeset ID	Gene Symbol	Gene name	logFC	t	*P*-value	adjusted *P*-value
1559949_at	TRPS1	Trichorhinophalangeal syndrome I	6.2601	18.9200	4.95E-23	5.16E-23
214329_x_at	TNFSF10	Tumor necrosis factor (ligand) superfamily, member 10	8.7780	26.8322	2.67E-29	3.37E-29
217782_s_at	GPS1	G protein pathway suppressor 1	6.5087	27.9268	4.90E-30	6.44E-30
219284_at	Hspbap1	HSPB (heat shock 27 kDa) associated protein 1	8.8694	39.2049	2.14E-36	5.23E-36
225823_at	C19orf70	Chromosome 19 open reading frame 70	7.5612	27.2640	1.36E-29	1.74E-29
230214_at	MRVI1	Murine retrovirus integration site 1 homolog	5.6772	27.4084	1.09E-29	1.40E-29
237588_at	SMCHD1	Structural maintenance of chromosomes flexible hinge domain containing 1	5.8131	26.7462	3.06E-29	3.85E-29
1556735_at	Unknown	Unknown	6.6352	25.8057	1.39E-28	1.69E-28

**Table 2:** KEGG enrichment analysis using the GATHER database.

#	Annotation	*P*-value	Bayes Factor	Genes (With Ann)	Genes (No Ann)	Genome (With Ann)	Genome (No Ann)	Genes
1	path:hsa04210: Apoptosis	0.0008	3	1	0	95	3034	TNFSF10
2	path:hsa04060: Cytokine-cytokine receptor interaction	0.0020	2	1	0	256	2873	TNFSF10

Genes (With Ann): the number of genes from the list with the annotation. Genes (No Ann): the number of genes from the list without the annotation. Genome (With Ann): the number of genes in the genome (excluding those in the list) with the annotation. Genome (No Ann): the number of genes in the genome (excluding those in the list) without the annotation. Genes: the symbols of the genes that have the annotation.

### A Robust Gene Expression Profile Classifier

As evaluated with a 10-fold cross-validation method in the whole dataset, the SVM model had the best discriminative ability with a predictive accuracy of around 86% (Table 3). The p predictive accuracy of SVM was higher than the figures of the rest models. In terms of the Sensitivity, Specificity and F1 score, SVM also outperformed the other models, as has been observed when compared with bio-inspired algorithms [17]–[20]. The Sensitivity, Specificity and F1 score for SVM reached over 92%, 78% and 89%, respectively. The R code for the feature selection algorithms and classification model building were provided (Table S3 in File S1.).

**Table 3:** Evaluation of multiple classification models including Support Vector Machine (SVM), Random Forest (RF), naïve Bayes (Bayes), Neural Network (NNT), K-Nearest Neighbor (KNN) and Logistic regression models via 10-fold cross-validation (10FCV).

Evaluation method	Model	Sensitivity	Specificity	F1score	Accuracy
*10FCV*	SVM	0.9231	0.7778	0.8889	0.8636
	RF	0.8462	0.7222	0.8302	0.7955
	naïve Bayes	0.6923	0.8889	0.7826	0.7727
	NNT	0.8846	0.7222	0.8519	0.8182
	KNN	0.8462	0.7222	0.8302	0.7955
	Logistic	0.7692	0.7778	0.8000	0.7727

## Discussion

At present, microarray technology is extensively used in biomedical research, and the data processing method is the key part for analyzing gene-chip results. Questions remain as to how to analytically deal with this type of data. The challenge is to detect a panel of discriminative genes from a large pool of candidate genes [21], [22]. To analyze the microarray data, this study proposed to integrate three feature ranking algorithms (SVM-RFE, ROC and Boruta) as the core into a combined algorithm. The combined algorithm generated an ordered gene set that consists of genes at a medium size. This work established a classification model for gene selection using multiple sclerosis gene expression data. The distinction between the three feature selection algorithms and the classification models was that the feature selection algorithms were used to detect a group of discriminative genes from a large number of candidates, reducing the dimensionality of data sets, and the models were built and assessed based on the selected genes for sample predictions. In evaluating the performance of different models, four measures including Sensitivity, Specificity, Accuracy and F1 score were calculated based on the confusion matrix output by each classifier using total dataset. Sensitivity (the true positive rate) measures the proportion of true positives which are correctly identified, and Specificity (the true negative rate) measures the proportion of negatives which are correctly identified. Accuracy and F1 score measures a model’s prediction accuracy rate. All the four statistics reach their best values at 1 and worst score at 0. We assessed the four statistics, and determined a relative optimal classifier with highest Sensitivity, Specificity, Accuracy and F1 score.

In this study, 8 genes were identified to be associated with multiple sclerosis. We built an SVM as the best model for sample prediction, having a predictive accuracy of around 86%. The SVM outperformed the other models as assessed by Sensitivity, Specificity, F1 score and Accuracy. The KEGG enrichment analysis suggested that the genes selected were statistically related to pathways involving apoptosis and cytokine-cytokine receptor interaction. Among the 8 genes, TNFSF10 had a close relationship with multiple sclerosis. Gene Ontology enrichment analysis revealed that TNFSF10 involved in the biological processes including protein kinase cascades, regulation of signal transduction and apoptosis, and the GPS1 and TRPS1 were primarily enriched in multiple sclerosis.

Apoptosis is a common regulatory mechanism for maintaining normal development and homeostasis of the immune system. Because the process of eliminating auto-reactive T cells via apoptosis is impaired in multiple sclerosis, apoptosis signaling-related genes may be strong candidate genes for involvement in multiple sclerosis [23]. According to the GeneCards database, there were six published studies [24]–[29] referring to the relationship between TNFSF10 and multiple sclerosis, indicating TNFSF10 might have an important role in multiple sclerosis. The increasing expression of TNFSF10 was observed in peripheral blood mononuclear cells of patients with multiple sclerosis. TNFSF10 belongs to the tumor necrosis factor/nerve growth factor superfamily [30], and can induce cell death or apoptosis of inflammatory cells. Blockade of TNFSF10 expressed in CD4+ myelin-specific T cells reduces caspase-dependent neuronal cell death in an experimental animal model for multiple sclerosis [31]. TNFSF10 involves both in cell death and other immunoregulatory mechanisms. According to Kikuchi et al. [24], the presence of the CC genotype in the coding region of TNFSF10 at position 1595 in exon 5 associated with a higher risk of multiple sclerosis in Japanese patients. Also, more than 80% of the top 30 most significant genes in multiple sclerosis were categorized into apoptosis signaling-related genes, and among them TNFSF10 was one of the significantly up-regulated genes [25]. In addition, a more recent candidate gene case-control study in the Spanish population finds an association of 3 SNPs in TRAIL, TRAILR-1 and TRAILR-2 genes with susceptibility to multiple sclerosis [32].

Besides TNFSF10, the rest 7 genes showed markedly differential expression between multiple sclerosis patients and controls, appearing to be functionally related to apoptosis. TRPS1 executes multiple functions in proliferating chondrocytes and activates proliferation in columnar cells according to the function annotations from the GeneCards database. TRPS1 was also suggested to be an apoptosis-associated gene that acts as a death-signaling gene to induce the elimination of cells via apoptosis [33]. GPS1 is known to suppress survival-associated mitogen-activated protein kinase-mediated signal transduction [34]–[38]. Hspbap1 is believed to inhibit the neuroprotective effects of heat shock protein 27, and is found extensively in the anterior temporal neocortex of patients with intractable epilepsy [39]. MRVI1 and SMCHD1 are respectively linked to blood coagulation and chromosome organization.

Several studies [8], [40]–[42] had explored gene expression patterns in multiple sclerosis. Brynedal et al. [4] evaluated the association between transcripts and group specificity using *t*-tests to detect differentially expressed genes, and estimated the fold change of genes between different groups. However, these studies identified a large amount of differentially regulated transcripts between different groups. Indeed, it is important to apply more effective approaches to analyze microarray data, where there are many thousands of features, and a few tens to hundreds of samples. Using the existing *t*-test approach to detect differentially expressed genes between samples always increases the discovery rate of false positive. Prior studies [5], [6] showed that combinatorial gene selection methods could be effectively applied to identify disease-related genes. Inspired by this idea, this work proposed a combinational strategy to predict multiple sclerosis samples using microarray data. Gene Ontology analysis in this study showed that the MAPK and protein kinase cascade signaling pathways were enriched in patients with multiple sclerosis, which was consistent with the results from Brynedal et al. [4].

This work performed a combined approach integrating feature ranking algorithms and an SVM classification model for gene selection. We can estimate the discriminative ability of each gene using the proposed approach, allowing an objective and quantitative evaluation of each gene. Due to the limitation that more gene expression profile datasets of multiple sclerosis cannot be available at present, other independent datasets are necessary to an appropriate validation of the algorithm in the future.

## Supporting Information

File S1 **File S1: Contains Tables S1-S3. Table S1. Gene Ontology analysis of the selected genes using GATHER (** http://gather.genome.duke.edu/ **). Table S2. The strength of association between genes and disease indicated as the counts of publications retrieved from GeneCards (until September 1, 2012).** Accordingly, more related studies retrieved by GeneCards supports much stronger association between genes and potential diseases. **Table S3. R code of feature selection algorithms and a robust SVM classification model.** Feature selection algorithms (SVM-RFE, ROC and Botuta) and classification models (SVM, Random Forests, naïve Bayes, Artificial Neural Network, Logistic Regression and k-Nearest Neighbor) were built within R software. The symbol of ‘#’ referred to the program annotation.
(DOC)
