# Supplementary material for: Mining Gene Expression Data of Multiple Sclerosis
Source: PLoS One. 2014 Jun 16;9(6):e100052. doi: 10.1371/journal.pone.0100052 (PMC4059716; doi:10.1371/journal.pone.0100052)
Supplement: File S1 — Contains Tables S1-S3. Table S1. Gene Ontology analysis of the selected genes using GATHER ( http://gather.genome.duke.edu/ ). Table S2. The strength of association between genes and disease indicated as the counts of publications retrieved from GeneCards (until September 1, 2012). Accordingly, more related studies retrieved by GeneCards supports much stronger association between genes and potential diseases. Table S3. R code of feature selection algorithms and a robust SVM classification model. Feature selection algorithms (SVM-RFE, ROC and Botuta) and classification models (SVM, Random Forests, naïve Bayes, Artificial Neural Network, Logistic Regression and k-Nearest Neighbor) were built within R software. The symbol of ‘#’ referred to the program annotation. (DOC) [file pone.0100052.s001.doc]

**Supporting Information File S1.**

**Table S1. Gene Ontology analysis of the selected genes using GATHER (http://gather.genome.duke.edu/).**

| # | Annotation | Bayes Factor | p Value | Genes | Genes (With Ann) | Genes (No Ann) | Genome (With Ann) | Genome (No Ann) |
| --- | --- | --- | --- | --- | --- | --- | --- | --- |
| 1 | GO:0007243 [6]: protein kinase cascade | 5 | 0.0002 | GPS1 TNFSF10 | 2 | 1 | 250 | 12026 |
| 2 | GO:0000188 [8]: inactivation of MAPK | 4 | 0.0003 | GPS1 | 1 | 2 | 12 | 12264 |
| 3 | GO:0007254 [8]: JNK cascade | 3 | 0.001 | GPS1 | 1 | 2 | 37 | 12239 |
| 4 | GO:0007242 [5]: intracellular signaling cascade | 3 | 0.002 | GPS1 TNFSF10 | 2 | 1 | 989 | 11287 |
| 5 | GO:0043123 [6]: positive regulation of I-kappaB kinase/NF-kappaB cascade | 3 | 0.003 | TNFSF10 | 1 | 2 | 78 | 12198 |
| 6 | GO:0000165 [7]: MAPKKK cascade | 3 | 0.003 | GPS1 | 1 | 2 | 80 | 12196 |
| 7 | GO:0043122 [5]: regulation of I-kappaB kinase/NF-kappaB cascade | 3 | 0.003 | TNFSF10 | 1 | 2 | 81 | 12195 |
| 8 | GO:0009967 [5]: positive regulation of signal transduction | 2 | 0.003 | TNFSF10 | 1 | 2 | 89 | 12187 |
| 9 | GO:0007249 [7]: I-kappaB kinase/NF-kappaB cascade | 2 | 0.004 | TNFSF10 | 1 | 2 | 108 | 12168 |
| 10 | GO:0012502 [7]: induction of programmed cell death | 2 | 0.004 | TNFSF10 | 1 | 2 | 122 | 12154 |
| 11 | GO:0006917 [8]: induction of apoptosis | 2 | 0.004 | TNFSF10 | 1 | 2 | 122 | 12154 |
| 12 | GO:0043068 [6]: positive regulation of programmed cell death | 2 | 0.005 | TNFSF10 | 1 | 2 | 130 | 12146 |
| 13 | GO:0043065 [7]: positive regulation of apoptosis | 2 | 0.005 | TNFSF10 | 1 | 2 | 129 | 12147 |
| 14 | GO:0009966 [4]: regulation of signal transduction | 2 | 0.006 | TNFSF10 | 1 | 2 | 169 | 12107 |
| 15 | GO:0042981 [6]: regulation of apoptosis | 1 | 0.009 | TNFSF10 | 1 | 2 | 249 | 12027 |
| 16 | GO:0043067 [5]: regulation of programmed cell death | 1 | 0.009 | TNFSF10 | 1 | 2 | 251 | 12025 |
| 17 | GO:0051242 [5]: positive regulation of cellular physiological process | 1 | 0.09 | TNFSF10 | 1 | 2 | 260 | 12016 |
| 18 | GO:0043119 [4]: positive regulation of physiological process | 1 | 0.01 | TNFSF10 | 1 | 2 | 336 | 11940 |
| 19 | GO:0012501 [5]: programmed cell death | 1 | 0.01 | TNFSF10 | 1 | 2 | 441 | 11835 |
| 20 | GO:0006915 [6]: apoptosis | 1 | 0.01 | TNFSF10 | 1 | 2 | 439 | 11837 |
| 21 | GO:0050791 [3]: regulation of physiological process | 1 | 0.01 | TNFSF10 TRPS1 | 2 | 1 | 2551 | 9725 |
| 22 | GO:0008219 [4]: cell death | 1 | 0.02 | TNFSF10 | 1 | 2 | 469 | 11807 |
| 23 | GO:0016265 [3]: death | 1 | 0.02 | TNFSF10 | 1 | 2 | 473 | 11803 |
| 24 | GO:0007267 [4]: cell-cell signaling | 1 | 0.02 | TNFSF10 | 1 | 2 | 537 | 11739 |
| 25 | GO:0007165 [4]: signal transduction | 1 | 0.02 | GPS1 TNFSF10 | 2 | 1 | 2824 | 9452 |
| 26 | GO:0050789 [2]: regulation of biological process | 1 | 0.02 | TNFSF10 TRPS1 | 2 | 1 | 2865 | 9411 |
| 27 | GO:0051244 [4]: regulation of cellular physiological process | 1 | 0.02 | TNFSF10 | 1 | 2 | 566 | 11710 |
| 28 | GO:0007049 [5]: cell cycle | 0 | 0.02 | GPS1 | 1 | 2 | 712 | 11564 |
| 29 | GO:0006955 [4]: immune response | 0 | 0.02 | TNFSF10 | 1 | 2 | 746 | 11530 |
| 30 | GO:0050794 [3]: regulation of cellular process | 0 | 0.02 | TNFSF10 | 1 | 2 | 791 | 11485 |
| 31 | GO:0007154 [3]: cell communication | 0 | 0.02 | GPS1 TNFSF10 | 2 | 1 | 3473 | 8803 |
| 32 | GO:0006952 [5]: defense response | 0 | 0.03 | TNFSF10 | 1 | 2 | 837 | 11439 |
| 33 | GO:0009607 [4]: response to biotic stimulus | 0 | 0.03 | TNFSF10 | 1 | 2 | 957 | 11319 |
| 34 | GO:0008283 [4]: cell proliferation | 0 | 0.03 | GPS1 | 1 | 2 | 1057 | 11219 |

**Table S2. The strength of association between genes and disease indicated as the counts of publications retrieved from GeneCards (until September 1, 2012). Accordingly, more related studies retrieved by GeneCards supports much stronger association between genes and potential diseases.**

| # | Authors | Article Title | Publication Year |
| --- | --- | --- | --- |
| 1 | Wandinger etc. | TNF-related apoptosis inducing ligand (TRAIL) as a potential response marker for interferon-beta treatment in multiple sclerosis. | 2003 |
| 2 | Weber etc. | Identification and functional characterization of a highly polymorphic region in the human TRAIL promoter in multiple sclerosis. | 2004 |
| 3 | Kikuchi etc. | TNF-related apoptosis inducing ligand (TRAIL) gene polymorphism in Japanese patients with multiple sclerosis. | 2005 |
| 4 | Satoh etc. | Microarray analysis identifies an aberrant expression of apoptosis and DNA damage-regulatory genes in multiple sclerosis. | 2005 |
| 5 | Weinstock etc. | Interferon-beta modulates bone-associated cytokines and osteoclast precursor activity in multiple sclerosis patients. | 2006 |
| 6 | Buttmann etc. | TRAIL, CXCL10 and CCL2 plasma levels during long-term Interferon-beta treatment of patients with multiple sclerosis correlate with  flu-like adverse effects but do not predict therapeutic response. | 2007 |

**Table S3. R code of feature selection algorithms and a robust SVM classification model.** Feature selection algorithms (SVM-RFE, ROC and Botuta) and classification models (SVM, Random Forests, naïve Bayes, Artificial Neural Network, Logistic Regression and k-Nearest Neighbor) were built within R software. The symbol of ‘#’ referred to the program annotation.

| **Description of R code** |
| --- |
| **#SVM-RFE Algorithm:**  library(e1071)  svmrfeFeatureRankingForMulticlass=function(x,y){  n=ncol(x)  survivingFeaturesIndexes=seq(1:n)  featureRankedList=vector(length=n)  rankedFeatureIndex=n  while(length(survivingFeaturesIndexes)>0){  # train the support vector machine  svmModel=svm(x[, survivingFeaturesIndexes], y, cost=10, cachesize=500, scale=F, type="C-classification", kernel="linear" )  # compute the weight vector  multiclassWeights=svm.weights(svmModel)  #compute ranking criteria  multiclassWeights=multiclassWeights * multiclassWeights  rankingCriteria=0  for(i in 1:ncol(multiclassWeights))rankingCriteria[i]=mean(multiclassWeights[,i])  # rank the features  (ranking=sort(rankingCriteria, index.return=TRUE)$ix)  # update feature ranked list  (featureRankedList[rankedFeatureIndex] = survivingFeaturesIndexes[ranking[1]])  rankedFeatureIndex=rankedFeatureIndex - 1  # eliminate the feature with smallest ranking criterion  (survivingFeaturesIndexes=survivingFeaturesIndexes[-ranking[1]])  cat(length(survivingFeaturesIndexes),"\n")}  return(featureRankedList)}  svm.weights<-function(model){  w=0  if(model$nclasses==2){  w=t(model$coefs)%*%model$SV  }else{  # compute start-index  start <- c(1, cumsum(model$nSV)+1)  start <- start[-length(start)]  calcw <- function (i,j) {  # ranges for class i and j:  ri <- start[i] : (start[i] + model$nSV[i] - 1)  rj <- start[j] : (start[j] + model$nSV[j] - 1)  # coefs for (i,j):  coef1 <- model$coefs[ri, j-1]  coef2 <- model$coefs[rj, i]  # return w values:  w=t(coef1)%*%model$SV[ri,]+t(coef2)%*%model$SV[rj,]  return(w)}  W=NULL  for (i in 1 : (model$nclasses - 1)){  for (j in (i + 1) : model$nclasses){  wi=calcw(i,j)  W=rbind(W,wi)  }  }  w=W  }  return(w)  }  # Calling the svmrfeFeatureRankingForMulticlass function with our dataset;  # The raw dataset was converted into an ‘AffyData’, which is an ‘ExpressionSet’ object:  # The ‘status’ variable corresponded to the category information of samples:  MexAs=exprs(AffyData)  status=c(rep(2,18),rep(1,18),rep(2,6))  featureRankedList=svmrfeFeatureRankingForMulticlass(t(MexAs),status)  fc=rownames(exprs(AffyDataf))[featureRankedList[1:1000]]  **#ROC Algorithm:**  AffyData$status=factor(c(rep(2,18),rep(1,18),rep(2,6)),labels=c("normal","disease"))  rocs=rowpAUCs(AffyData,"status",p=0.2)  j=which(area(rocs)>=0.05)  jj=featureNames(AffyData)[j]  pAUC_s=sort(area(rocs[jj]),decreasing=TRUE)  pAUC_s_s=data.frame(pAUC_s[1:1000])  roc_f=rownames(pAUC_s_s)  **#Boruta Algorithm:**  library(Boruta)  MexAs=t(exprs(AffyData))  MexAsD=data.frame(MexAs)  MexAsD$status=c(rep(2,18),rep(1,18),rep(2,6))  set.seed(2012)  Boruta.all<-Boruta(status~.,data=MexAsD,doTrace=2,ntree=500,maxRuns=1000)  aB=attStats(Boruta.all)  aB_con=aB[which(aB$decision=="Confirmed"),][,c(1,6)]  aB_conM=as.matrix(aB_con)  aB_M=aB_conM[order(aB_conM[,1],decreasing=TRUE),]  B_f=rownames(aB_M)  B_f=substr(B_f,2,25)  **#Integrating three feature selection algorithms:**  sl=0  for (i in 1:1000){  for(j in 1:1000){  for (g in 1:length(B_f)){  if (fc[i]==roc_f[j]& roc_f[j]==B_f[g]) sl=c(sl,fc[i])  else sl=sl  }  }  }  sl=sl[2:length(sl)]  Section 2: In this section, we demonstrated the code for a robust SVM classification model for gene selection using gene expression microarray data. This proposed SVM model could be useful to select genes in multiple sclerosis and other diseases.  **#Building and assessing SVM model:**  library(MLInterfaces)  # The ‘TestInd’ and ‘TrainInd’ respectively corresponds to the testing and training datasets.  # 10-fold Cross-validation for the whole dataset:  SubAff0=AffyData[sl]  SubAff0$status=factor(c(rep(2,20),rep(1,18),rep(2,6)),labels=c("normal","disease"))  set.seed(2012)  svm1=MLearn(status~.,data=SubAff0,svmI,xvalSpec("LOG",10,balKfold.xvspec(10)))  cfp1=confuMat(svm1)  # Computing the metrics of Sensitivity, Specificity, Accuracy and F1 socre:  Sn=cfp1[2,2]/(cfp1[2,1]+cfp1[2,2])  Sp=cfp1[1,1]/(cfp1[1,1]+cfp1[1,2])  Ac=(cfp1[1,1]+cfp1[2,2])/sum(cfp1)  F1_score=2*cfp1[2,2]/(2*cfp1[2,2]+cfp1[2,1]+cfp1[1,2])  # 10-fold Cross-validation for the testing dataset:  set.seed(2012)  svm2=MLearn(status~.,data=SubAff0,svmI,trainInd=TrainInd)  cfp2_1=confuMat(svm2,"test")  Sn=cfp2_1[2,2]/(cfp2_1[2,1]+cfp2_1[2,2])  Sp=cfp2_1[1,1]/(cfp2_1[1,1]+cfp2_1[1,2])  Ac=(cfp2_1[1,1]+cfp2_1[2,2])/sum(cfp2_1)  F1_score=2*cfp2_1[2,2]/(2*cfp2_1[2,2]+cfp2_1[2,1]+cfp2_1[1,2])  # 10-fold Cross-validation for the training dataset:  SubAff1=AffyData[sl,TrainInd]  SubAff1$status=factor(c(rep(2,18),rep(1,14),rep(2,3)),labels=c("normal","disease"))  set.seed(2012)  svm3=MLearn(status~.,data=SubAff1,svmI,xvalSpec("LOG",10,balKfold.xvspec(10)))  cfp3=confuMat(svm3)  Sn=cfp3[2,2]/(cfp3[2,1]+cfp3[2,2])  Sp=cfp3[1,1]/(cfp3[1,1]+cfp3[1,2])  Ac=(cfp3[1,1]+cfp3[2,2])/sum(cfp3)  F1_score=2*cfp3[2,2]/(2*cfp3[2,2]+cfp3[2,1]+cfp3[1,2])  **#Prediction based on the SVM model:**  # ‘NewData’ corresponded to a new dataset:  NewData0=NewData[sl]  MyExp0=exprs(NewData0)  MyExp0=as.data.frame(MyExp0)  SubAff0=AffyData[sl]  SubAff0$status=factor(c(rep(2,20),rep(1,18),rep(2,6)),labels=c("normal","disease"))  set.seed(2012)  svm1=MLearn(status~.,data=SubAff0,svmI)  predict(svm1, MyExp0) |
